# Identification of CXCL10 and CXCL11 as the candidate genes involving the development of colitis-associated colorectal cancer

**DOI:** 10.3389/fgene.2022.945414

**Published:** 2022-08-08

**Authors:** Can Lu, Xiaopeng Zhang, Yang Luo, Jingang Huang, Minhao Yu

**Affiliations:** ^1^ Faculty of Medicine, Ludwig-Maximilians University, Munich, Germany; ^2^ Department of Colorectal Surgery, The Second Affiliated Hospital of Zhejiang University School of Medicine, Hangzhou, China; ^3^ School of Medicine, Technical University of Munich, Munich, Germany; ^4^ Gastrointestinal Cancer Center, Key Laboratory of Carcinogenesis and Translational Research (Ministry of Education), Peking University Cancer Hospital and Institute, Beijing, China; ^5^ Department of Gastrointestinal Surgery, Renji Hospital, School of Medicine, Shanghai Jiaotong University, Shanghai, China; ^6^ Guangdong Provincial Key Laboratory of Malignant Tumor Epigenetics and Gene Regulation, Guangdong-Hong Kong Joint Laboratory for RNA Medicine, Sun Yat-Sen Memorial Hospital, Sun Yat-Sen University, Guangzhou, China; ^7^ Medical Research Center, Sun Yat-Sen Memorial Hospital, Sun Yat-Sen University, Guangzhou, China

**Keywords:** ulcerative colitis, colorectal cancer, colitis-associated colorectal cancer, differentially expressed genes, TCGA

## Abstract

**Background:** Ulcerative colitis (UC) is a well-known risk factor for developing colitis-associated colorectal cancer (CAC). However, the molecular mechanism of the pathogenesis of CAC remains unclear. This study aimed to explore candidate genes involved in the tumorigenesis of CAC.

**Methods:** GSE75214 and the Cancer Genome Atlas Program (TCGA) dataset were used to analyze the differentially expressed genes (DEGs) in UC and colorectal cancer (CRC), respectively. Survival-hub genes were identified from these DEGs by sequentially constructing a protein–protein interaction network, selecting hub genes, and conducting survival analysis. Regulatory signatures were also predicted on these genes through the online database. *Apc* ^*min/+*^ and UC mice models were used to validate the expression of the above-predicted molecules. Gene set enrichment analysis and CIBERSORT were performed to explore the enriched molecular pathways and associated tissue-infiltrating immune cells of genes.

**Results:** Here, 376 common DEGs were identified from the GSE75214 and TCGA datasets. Through survival-hub gene selection and *in vivo* experiments, we confirmed that CXCL10 and CXCL11 were significantly upregulated in UC and CRC. We also proved that miR-34a-5p and miR-203a-5p were potential regulators of CXCL10 and CXCL11. Meanwhile, CXCL10 and CXCL11 may activate the JAK–STAT signaling pathway via the interaction with cytokine receptors in UC. Furthermore, CXCL10 and CXCL11 were positively associated with the tissue infiltration of proinflammatory M1 macrophages in UC and CRC.

**Conclusion:** CXCL10 and CXCL11 may act as the candidate genes involved in the tumorigenesis of CAC and potential therapeutic targets to prevent the development of CAC from UC.

## Introduction

Ulcerative colitis (UC) is a chronic inflammatory bowel disorder characterized by relapsing and remitting mucosal inflammation that starts in the rectum and generally extends proximally through the colon in a continuous manner (Ungaro et al., 2017). Although UC incidence has stabilized in western countries since 1990, the worldwide incidence and prevalence of this disease are greatly increasing as emerging industrialized societies have adopted a more westernized lifestyle (Ng et al., 2017). The highest prevalence rates of UC have been reported in Europe (505 per 100,000 in Norway) and North America (286 per 100,000 in the United States) (Ng et al., 2017). So far, the precise pathogenesis of UC remains unclear, but genetic susceptibility, dysregulated immune system, microbial dysbiosis, and environmental exposure are all potential pathogenic factors (Du and Ha, 2020). The peak age of UC onset is between the third and fourth decades of life without sex predominance (Cosnes et al., 2011), which tremendously affects patients’ productivity and imposes an immersive financial burden on health systems.

UC is a critical risk factor for colorectal cancer (CRC) development. Although the overall risk of CRC in patients with UC is not different from that of the general population, at least in the first decade after diagnosis, those with long-duration extensive colitis or those diagnosed with UC at a young age remain at a significantly increased risk of CRC development (Jess et al., 2012b). A meta-analysis also revealed that the cumulative risk of CRC could reach 13.9% in patients with a 30-year duration of UC onset (Bopanna et al., 2017). Although colitis-associated CRC (CAC) originating from UC patients only takes up approximately 1% of all CRC cases, one-sixth of all deaths in UC patients were caused by CAC (Gyde et al., 1982). Therefore, it is imperative to deepen our understanding of the cumulative detrimental effects of UC and to develop new agents to impede the occurrence of CAC. However, the molecular mechanism of CAC development remains unknown.

In UC, chronic inflammation is knowingly associated with the pathogenesis of CAC via the production of inflammatory mediators, oxidative stress, and alterations in immune receptor expression on epithelial cells (Kusunoki, 2015). To highlight the impact of the inflammation on intestine tissues, we only enrolled datasets containing UC patients with an active inflammation status. Meanwhile, the Cancer Genome Atlas Program (TCGA) projects provide the largest repository of expression matrices for CRC patients and paracancerous controls at the single-dataset level. We performed a series of bioinformatics analyses in the present study to identify survival-hub genes, including differential expression analysis, protein–protein interaction network, selection of hub genes, and survival analysis. Furthermore, we predicted the regulatory signatures on these genes using the online database. Moreover, we used *in vivo* experiments to validate the expression difference of survival-hub genes and regulatory signatures in both UC and CRC mice models. Our study demonstrated that CXCL10 and CXCL11 were candidate genes involved in the pathogenesis of CAC, indicating that targeting CXCL10/11 is a promising therapeutic strategy. To our knowledge, this is the first study to explore the underlying carcinogenic mechanism of CAC development using bioinformatics and animal models.

## Materials and methods

### Data collection and processing

The Gene Expression Omnibus database was thoroughly searched to find eligible UC datasets with the following inclusion criteria: 1) UC patients with an active inflammation status, 2) a UC group with more than 20 patients, and 3) gene expression profiles based on tissue samples. GSE75214 (Vancamelbeke et al., 2017) containing 74 UC patients with an active inflammation status and 11 healthy controls were enrolled for UC analysis. *GEOquery* (RRID: SCR 000146) R package was used to download the expression matrices of this dataset. The probes were annotated into gene symbols based on the GPL6244 annotation files. When multiple probes matched one gene, the median was calculated as its expression values. Gene expression profiles of 568 CRC patients and 51 healthy controls were downloaded from TCGA through the GDC data portal. Clinical follow-up data of these patients were acquired from the University of Santa Cruz Xena platform.

### Differential expression analysis

Differential expression analysis in the GSE75214 and TCGA cohorts was conducted using the *limma* (RRID: SCR_010943) and *DESeq2* (RRID: SCR_015687) R packages, respectively. Any gene with adjusted *p* values of <0.01 and |log2(Foldchange)| of >1 was regarded as differentially expressed genes (DEGs). DEGs consistently changed in the above two datasets were identified as common DEGs.

### Functional enrichment analysis

To determine the potential function of the identified common DEGs, we used the *clusterProfiler* (RRID: SCR_016884) R package to carry out gene ontology (GO) and Kyoto Encyclopedia of Genes and Genomes (KEGG) pathway analyses. GO analysis was divided into three categories: biological process (BP), molecular function (MF), and cellular components (CC). The cutoff criteria of *p* values <0.05 and false discovery rate <0.05 were regarded as statistically significant differences for all analyses.

### Protein–protein interaction networks

The online database Search Tool for the Retrieval of Interacting Genes (STRING, version 11.0, RRID: SCR_005223) (Szklarczyk et al., 2019) was used to evaluate the interactive relationships among common DEGs. Just the interaction pairs with a combined score of >0.7 were selected. Then, Cytoscape software (version 3.8.2, RRID: SCR_003032) was used to construct and visualize a protein–protein interaction (PPI) network of DEGs (Shannon et al., 2003). The cytoHubba (RRID: SCR_017677) plugin was applied to define the top 10 hub genes of the network using the maximal clique centrality method of topological analysis.

### Survival analysis

We applied the Kaplan–Meier plot to analyze the overall survival (OS) and progression-free interval (PFI) probability of different groups using two R packages, namely, *survival* (RRID: SCR_021137) and *survminer* (RRID: SCR_021094). OS represented the interval from the diagnosis date until the date of death from any cause, and PFI referred to the interval from the diagnosis date until the date of the first occurrence of a new tumor event, including the progression of the disease, locoregional recurrence, distant metastasis, new primary tumor, or death with tumor (Liu et al., 2018).

### Identification of regulatory signatures interacted with genes

Transcription factor–gene and miRNA–gene interactions were analyzed to identify transcription factors (TFs) and miRNAs that regulate the expression of genes at the transcription and posttranscription levels, respectively. JASPAR (RRID: SCR_003030) is an open-access database of curated, nonredundant TF binding profiles stored as position frequency matrices and TF flexible models for TFs across multiple species in taxonomic groups (Stormo, 2013). Moreover, TarBase (version 8.0, RRID: SCR_000577) is one of the largest databases of miRNA–target interactions with experimental support (Karagkouni et al., 2018). NetworkAnalyst (version 3.0, RRID: SCR_016909) was applied to predict potential TFs and miRNAs of genes from the JASPAR and TarBase databases, respectively. Then, Cytoscape software was used to visualize the TF–gene and miRNA–gene interaction networks.

### Mice

*Apc* ^*min/+*^ mice were purchased from Jackson Laboratory, and C57BL/6 (MGI Cat# 2159769, RRID: MGI:2159769) mice were obtained from SLAC Laboratory Animal Co., Ltd. (Shanghai, China). The mice were maintained in a pathogen-free animal facility, and all experiments were performed in mice aged 9–14 weeks. All animal experiments were performed according to the National Institute of Health Guidelines for the Care and Use of Laboratory Animals. Our study was approved by the Animal Care and Use Committee of Renji Hospital, School of Medicine, Shanghai Jiao Tong University. At the end of the experiment, under inhalation anesthesia with isoflurane, mice were sacrificed by strangulating their neck, and then, their intestine was harvested for further analysis.

### Acute dextran sulfate sodium-induced colitis mouse model

Acute colitis was induced in C57BL/6J mice with the administration of 3% dextran sulfate sodium (DSS) with a molecular mass of 40 kDa (Sigma Aldrich, Darmstadt, Germany) in autoclaved drinking water. After acclimation, 8-week-old mice were randomly divided into two groups (*n* = 6 per group): 0 DSS (negative control) and 3% DSS-treated group. Mice were treated with 3% DSS for 6 days and plain water for 3 days right after the treatment. The severity of colitis was assessed daily by measuring weight loss and disease activity index (DAI). DAI was calculated based on the degree of diarrhea and visible fecal blood as Cooper et al. described (Cooper et al., 1993). Mice were euthanized on day 10. The intestine was removed and meshed for further analysis.

### Western blot assay

The freshly removed intestines were meshed and lysed with RIPA lysis buffer (Thermo Fisher Scientific, Waltham, MA, United States) on ice. The protein concentrations were measured with Bradford assay (Bio-Rad, Hercules, CA, United States), and 20 µg of protein per sample was subjected to 10% SDS-acrylamide gels for electrophoresis. The proteins were separated by electrophoresis at 80–120 V in an electrophoresis unit (Invitrogen, Waltham, MA, United States) with NuPAGE™ MOPS SDS as a running buffer. The separated proteins were transferred onto Immobilon PVDF membranes (Invitrogen, Waltham, MA, United States) with NuPAGE™ Transfer buffer using the Invitrogen blotting system and a BIO-RAD power supply constantly held at 125 mA and a maximum voltage of 10 V. After blocking in 5% skimmed milk/TBS–Tween 20, the membrane was incubated with a primary antibody and then with horseradish-peroxidase (HRP)-conjugated secondary antibodies. Enhanced chemiluminescence (Thermo Fisher Scientific, Waltham, MA, United States) signals were recorded using a 440-CF imaging system (Kodak, Rochester, NY, United States). Primary antibodies included mouse antiactin antibody (Santa Cruz Biotechnology Cat# sc-8432, RRID: AB_626630) and rabbit anti-YY1 antibody (Abcam, Cambridge, Cat# ab245365).

### RNA extraction and qRT-PCR

Total RNA was extracted from cells using the NucleoZOL reagent (MACHEREY-NAGEL, Düren, Germany) according to the manufacturer’s instructions. Here, 2 µg of total RNA was reverse-transcribed using SuperScript III First-Strand Synthesis SuperMix for qPCR (Thermo Fisher Scientific, Waltham, MA, United States). For the detection of miRNAs, a TaqMan MicroRNA Reverse Transcription Kit (Thermo Fisher Scientific, Waltham, MA, United States) was applied for the synthesis of cDNAs. Each cDNA sample was similarly diluted for subsequent PCR amplification with the 2× qPCR Master Mix (Sigma Aldrich, Darmstadt, Germany) with a StepOnePlus Real-Time PCR System (Thermo Fisher Scientific, Waltham, MA, United States). The expression of miRNAs was detected with a TaqMan MicroRNA Assay (Thermo Fisher Scientific, Waltham, MA, United States). The qPCR results were calculated using the 2^−ΔΔCt^ method. Results were represented as fold induction of the disease condition compared with the control condition. All primers used in this study are presented in Supplementary Table S1.

### Cytokine array

Intestines from all mice models were collected and cut into pieces. After overnight incubation in Hank’s Balanced Salt Solution (Thermo Fisher Scientific, Waltham, MA, United States), tissues were removed through a 350 g centrifuge, and the supernatant was subjected to the proteome profiler mouse XL cytokine array (R&D system, Minneapolis, MN, United States). Signals were visualized using an myECL imager or the iBright imaging system (Thermo Fisher Scientific, Waltham, MA, United States).

### Gene set enrichment analysis

We applied gene set enrichment analysis (GSEA) (Subramanian et al., 2005) to predict the KEGG pathways related to CXCL10 and CXCL11 using the *clusterProfiler* R package with the following parameters: minGSSize = 10, maxGS-Size = 500, nPerm = 100, seed = 2020, and *p-*value corrected by Benjamini–Hochberg (BH). Significant enrichment terms were considered if the adjusted *p*-value was less than 0.05. The KEGG gene sets were downloaded from the MSigDB database (https://www.gsea-msigdb.org).

### Evaluation of immune cell infiltration

The CIBERSORT algorithm (Chen et al., 2018) was used to estimate the proportions of 22 immune cells in UC and CRC samples. Spearman’s correlation was calculated between the proportions and gene expression. *p* < 0.05 was considered a significant correlation.

### Statistical analysis

The log-rank test was used to evaluate the survival difference between different groups in the Kaplan–Meier plot. The statistical difference between the two groups was calculated using the Wilcoxon rank-sum test or *t*-test, and a *p*-value of <0.05 was regarded as the significant threshold. Statistical analyses were conducted using R software (version 4.0.5).

## Results

### Identification of overlapping differentially expressed genes across ulcerative colitis and colorectal cancer

**FIGURE 1:**
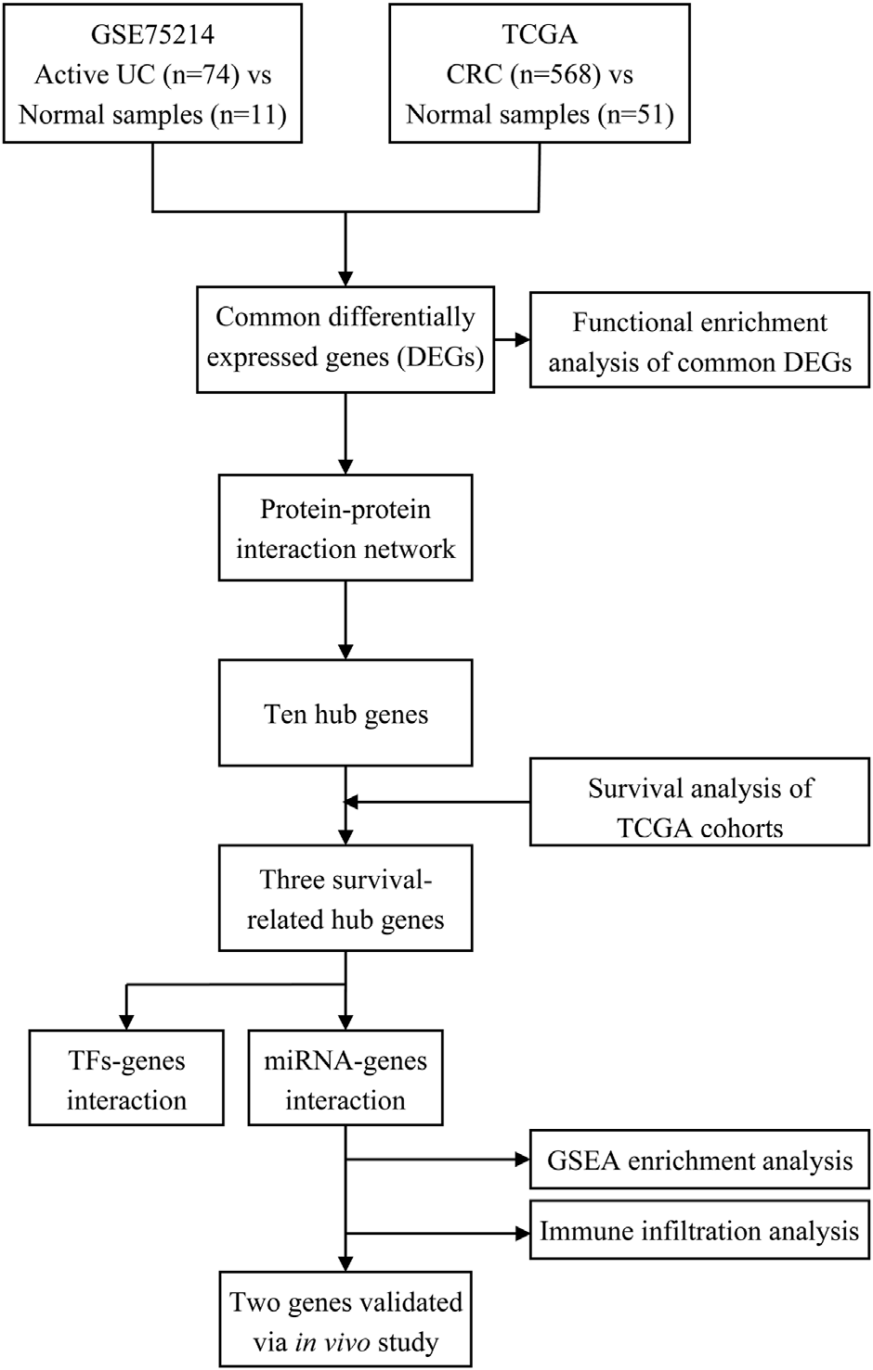
Workflow of processing the datasets. Abbreviation: UC, ulcerative colitis; TCGA, The Cancer Genome Atlas; CRC, colorectal cancer; TFs, transcription factors; miRNA, microRNA.

**FIGURE 2:**
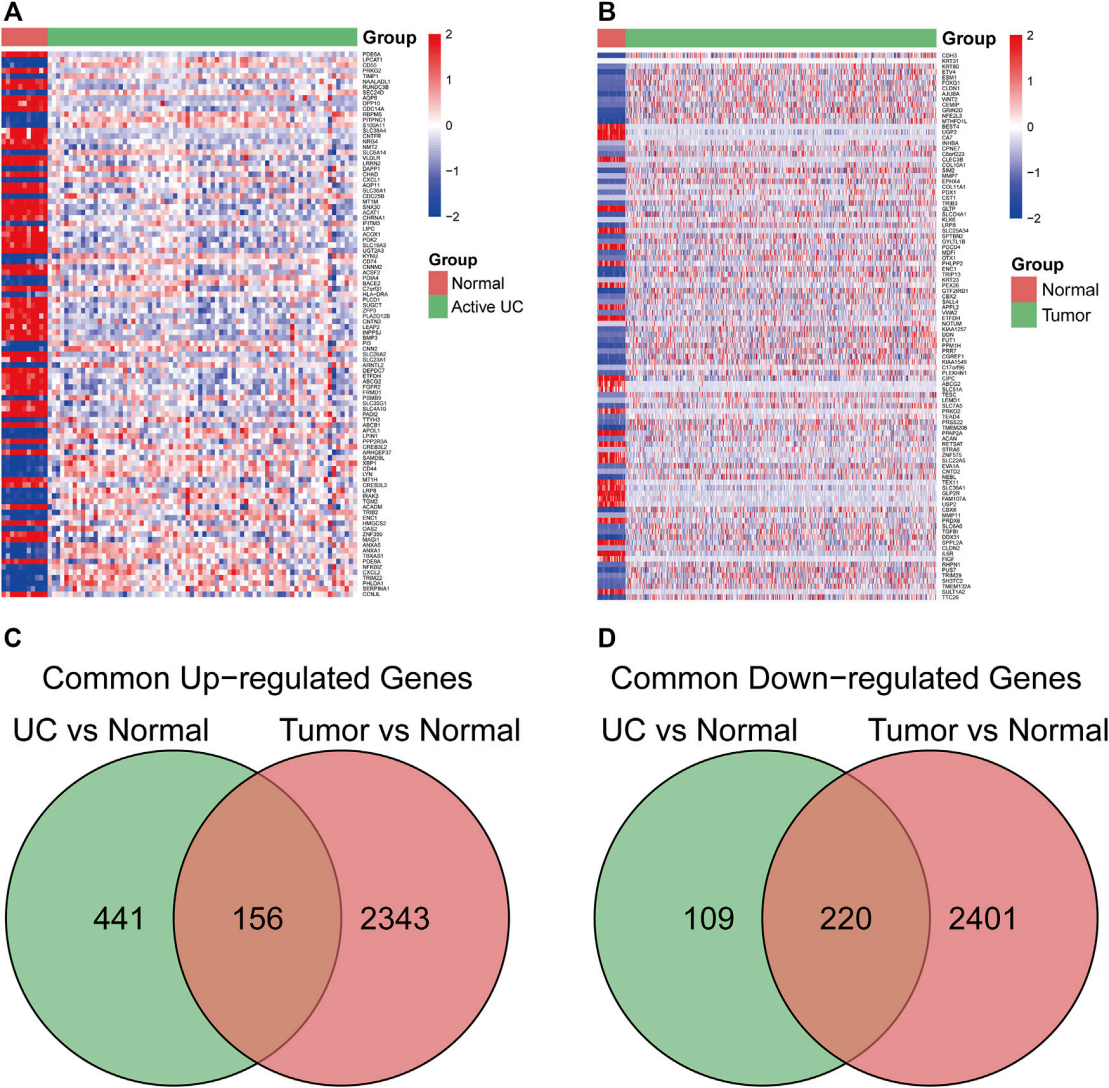
The common differentially expressed genes (DEGs) between ulcerative colitis (UC) and colorectal cancer (CRC). **(A)** heatmap of the top 100 DEGs in the UC dataset. **(B)** heatmap of top 100 DEGs in the CRC dataset. **(C)** common upregulated DEGs in UC and CRC. **(D)** common downregulated DEGs in UC and CRC. Abbreviation: DEGs, Differentially expressed genes; UC, ulcerative colitis; CRC, colorectal cancer.

The study flowchart is illustrated in Figure 1. In the GSE75214 dataset, we identified 926 DEGs in UC samples compared to healthy controls, including 597 upregulated genes and 329 downregulated genes. Meanwhile, 5120 DEGs were obtained from the differential expression analysis between CRC samples and noncancerous tissues in the TCGA cohort. The top 100 DEGs in the UC and CRC groups are displayed in Figures 2A,B. To dissect the underlying mechanisms involved in the malignant transformation of UC, we used the Venn diagram to intersect the consistent DEGs between the GSE75214 and TCGA cohorts. In total, there were 376 common DEGs consisting of 156 upregulated DEGs and 220 downregulated DEGs (Figures 2C,D).

### Functional enrichment analysis and protein–protein interaction network of common differentially expressed genes

**FIGURE 3:**
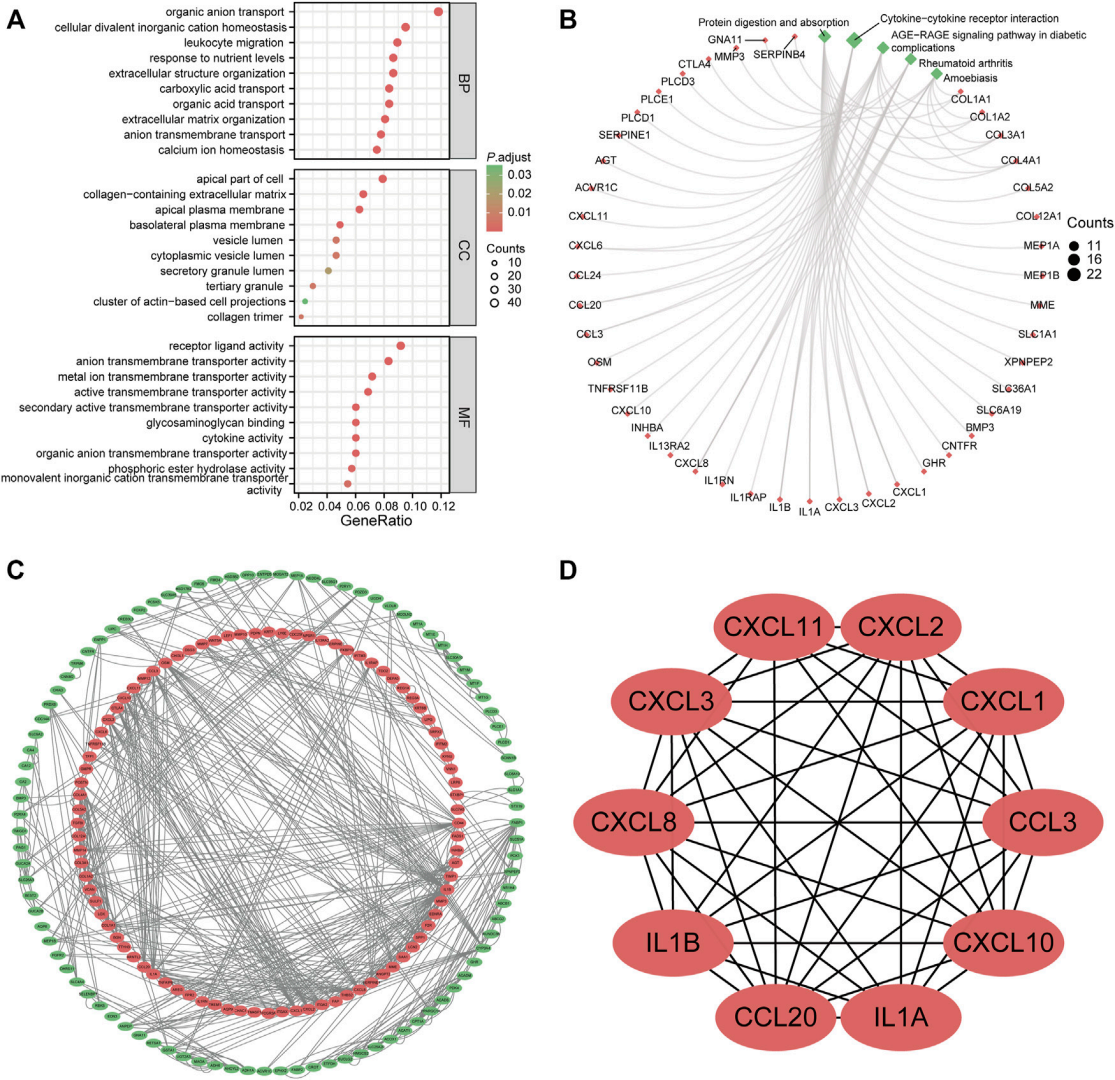
Functional enrichment analysis and protein–protein interaction (PPI) network of common differentially expressed genes (DEGs). **(A)** gene ontology enrichment analysis of common DEGs. **(B)** Kyoto Encyclopedia of Genes and Genomes enrichment analysis of common DEGs. **(C)** PPI network of common DEGs. The red node represented upregulated genes; the green node stands for downregulated genes. **(D)** hub genes identified from the PPI network. Abbreviation: DEGs, differentially expressed genes; BP, biological process; CC, cellular components; MF, molecular function; PPI, protein–protein interaction.

We performed GO and KEGG enrichment analyses to explore the biological functions of the shared DEGs between UC and CRC. These DEGs were mainly involved in transporting organic substances and leukocyte chemotaxis in three subtypes of GO terms (Figure 3A). Likewise, protein digestion and absorption and cytokine–cytokine receptor interaction were the principally enriched KEGG pathways of common DEGs (Figure 3B). Detailed results of the functional enrichment analysis are shown in Supplementary Table S2.

To identify the potential interactions of 376 DEGs, we constructed the PPI network based on the STRING database with the threshold of minimum required interaction score of >0.7. A total of 183 nodes and 590 edges were incorporated into this network, as shown in Figure 3C. Each node represented one gene, and the edges indicated the predicted interaction relationships. Furthermore, we used the cytoHubba plugin of Cytoscape software to identify hub genes from the whole PPI network. The top 10 genes are shown in Figure 3D, namely, CXCL1, CXCL2, CXCL3, CXCL8, CXCL10, CXCL11, CCL3, CCL20, IL1B, and IL1A.

### Survival analysis of hub genes in the Cancer Genome Atlas Program cohorts

**FIGURE 4:**
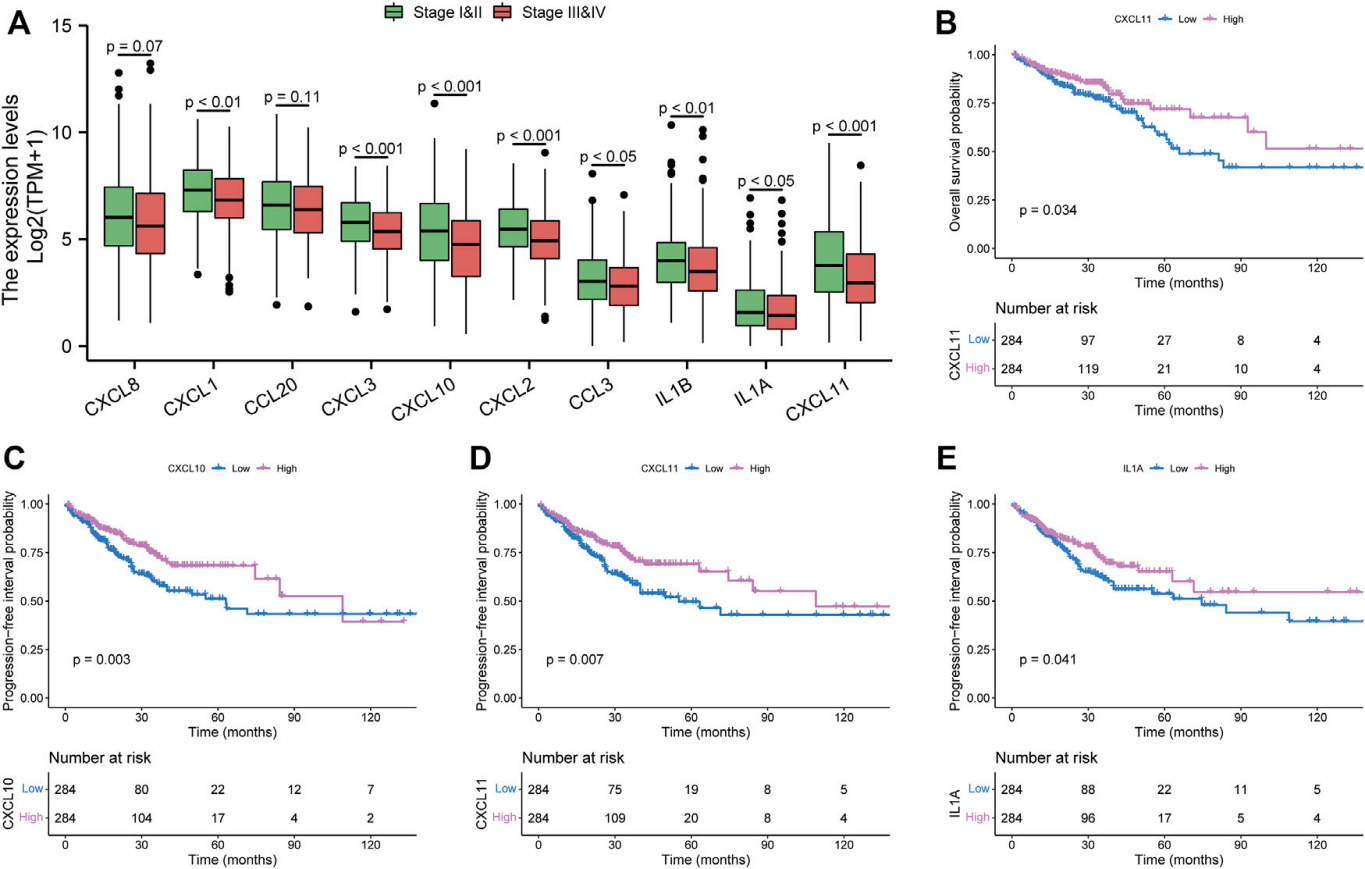
Clinical significance of hub genes in the Cancer Genome Atlas Program cohorts. **(A)** expression analysis of hub genes in different tumor stages. **(B)** overall survival analysis of CXCL11. **(C–E)** progression-free interval analysis of CXCL10, CXCL11, and IL1A, respectively.

To refine the clinical significance of hub genes, we used TCGA cohorts to analyze their expression difference stratified by tumor stage. As shown in Figure 4A, except for CXCL8 and CCL20, all hub genes have significantly lower expression in advanced tumor stages. Then, we further explored the prognostic effects of these hub genes in CRC patients. Just CXCL11 was associated with the OS in the TCGA cohort (Figure 4B). Meanwhile, three genes have a significant association with PFI (Figures 4C–E). In total, we only have three survival-related hub genes in this study, namely, CXCL10, CXCL11, and IL1A. Furthermore, survival analysis of other hub genes is shown in Supplementary Figure S1. The characteristics of the CRC patients are displayed in Supplementary Table S3.

### Regulatory signatures of survival-associated hub genes

**FIGURE 5:**
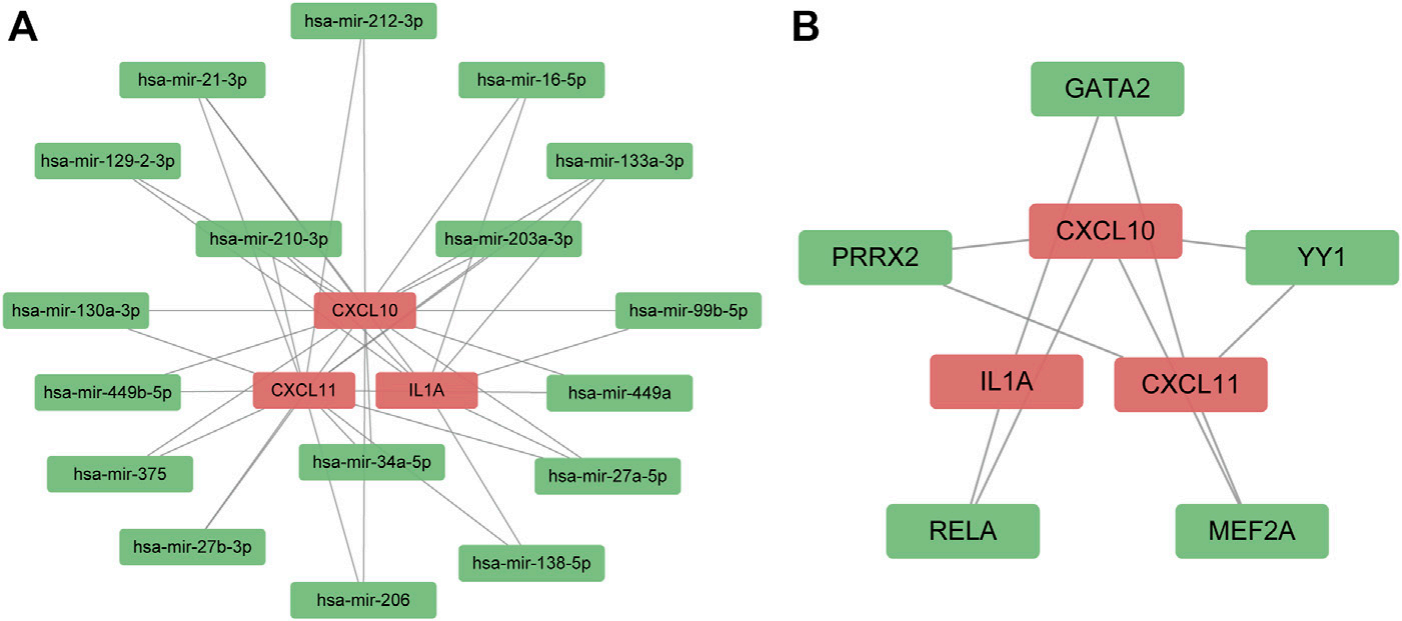
Regulatory signatures of survival-associated hub genes. **(A)** miRNA–gene interaction network; **(B)** transcription factors–gene interaction network. The red node represented survival-related hub genes. The green node indicated miRNAs and transcription factors.

Furthermore, we used the online database to predict TFs and miRNAs that might interact with survival-associated hub genes at the transcription and posttranscription levels. The medium degree cutoff was applied to reduce redundant nodes of the interaction networks. As shown in Figure 5A, the miRNAs-hub genes interaction network contained 17 miRNAs and 38 edges. Meanwhile, there were five TFs and 10 edges in the TF-hub gene network (Figure 5B).

### Validation of the expression of survival-hub genes and related regulatory signatures using the *in vivo* experiments

**FIGURE 6:**
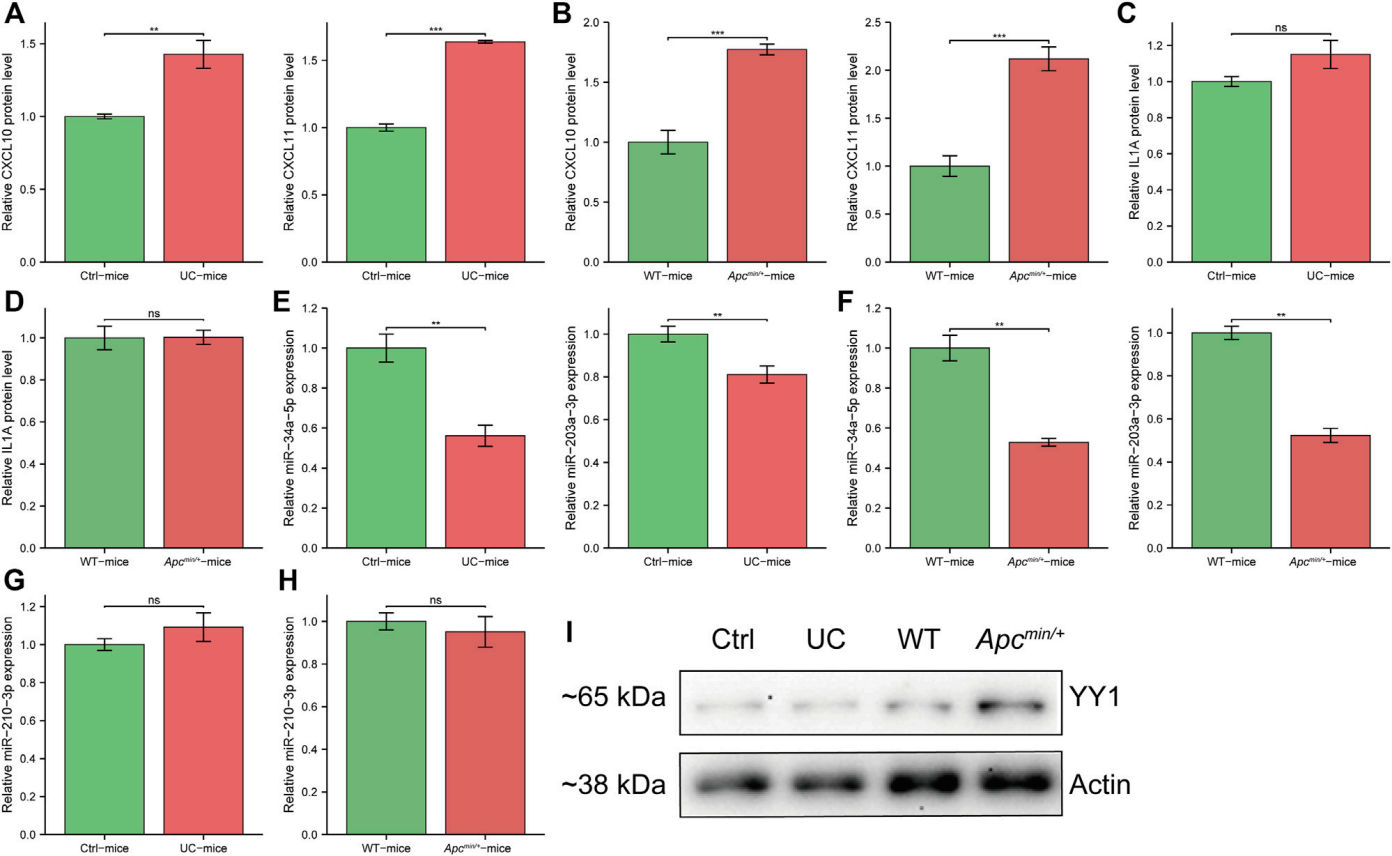
The expression of survival-hub genes and interacted regulatory signatures *in vivo*. **(A)** cytokine array to detect CXCL10 and CXCL11 in ulcerative colitis (UC) mice and controls (*n* = 3, per group), respectively. **(B)** cytokine array to detect CXCL10 and CXCL11 in *APC* ^*min/+*^ mice and wild type (WT) (*n* = 3, per group), respectively. **(C)** cytokine array to detect IL1A in UC mice and controls (*n* = 3, per group). **(D)** cytokine array to detect IL1A in *APC* ^*min/+*^ mice and WT (*n* = 3, per group). **(E)** qRT-PCR for miR-34a-5p and miR-203a-3p in UC mice and controls (*n* = 6, per group), respectively. **(F)** qRT-PCR for miR-34a-5p and miR-203a-3p in *APC* ^*min/+*^ mice and WT (*n* = 6, per group), respectively. **(G)** qRT-PCR for miR-210-3p in UC mice and controls (*n* = 6, per group). **(H)** qRT-PCR for miR-210-3p in *APC* ^*min/+*^ mice and WT (*n* = 6, per group). **(I)** Western blot for YY1 expression in different groups. Ctrl, control mice for UC model. WT, wild type mice. *, *p* < 0.05; **, *p* < 0.01, ***, *p* < 0.001. Abbreviation: Ctrl, control; UC, ulcerative colitis; WT, wild type.

To validate the upregulated expression of three survival-hub genes in UC and CRC samples, we performed cytokine array studies to detect the expression levels of these genes in UC and CRC mice models. We found that CXCL10 and CXCL11 were consistently upregulated in UC and CRC compared with the corresponding controls (Figures 6A,B), whereas there was no difference in IL1A expression (Figures 6C,D). Thus, CXCL10 and CXCL11 were regarded as the candidate genes involved in the pathogenesis of CAC. We selected three miRNAs possibly regulating CXCL10 and CXCL11 from the miRNA–gene network to examine the potential regulatory miRNAs further. Our results showed that miR-34a-5p and miR-203a-5p have significantly lower expression in UC and CRC than in controls (Figures 6E,F). However, miR-210-3p has a similar expression between the disease group and the controls (Figures 6G,H). Also, we detected the potential TFs of CXCL10 and CXCL11 in UC and CRC mice models. Several studies have reported that YY1 could promote the tumor progression of CRC (Fang et al., 2019; Tang et al., 2019; Yu et al., 2020). Our results indicated that YY1 was only upregulated in the CRC model (Figure 6I).

These results indicated that miR-34a-5p and miR-203a-5p might inhibit the tumorigenesis of the UC mucosa by mediating the downregulation of CXCL10 and CXCL11. Moreover, YY1 may not affect the development of CAC.

### Immune cell infiltration and gene set enrichment analysis of survival-hub genes

**FIGURE 7:**
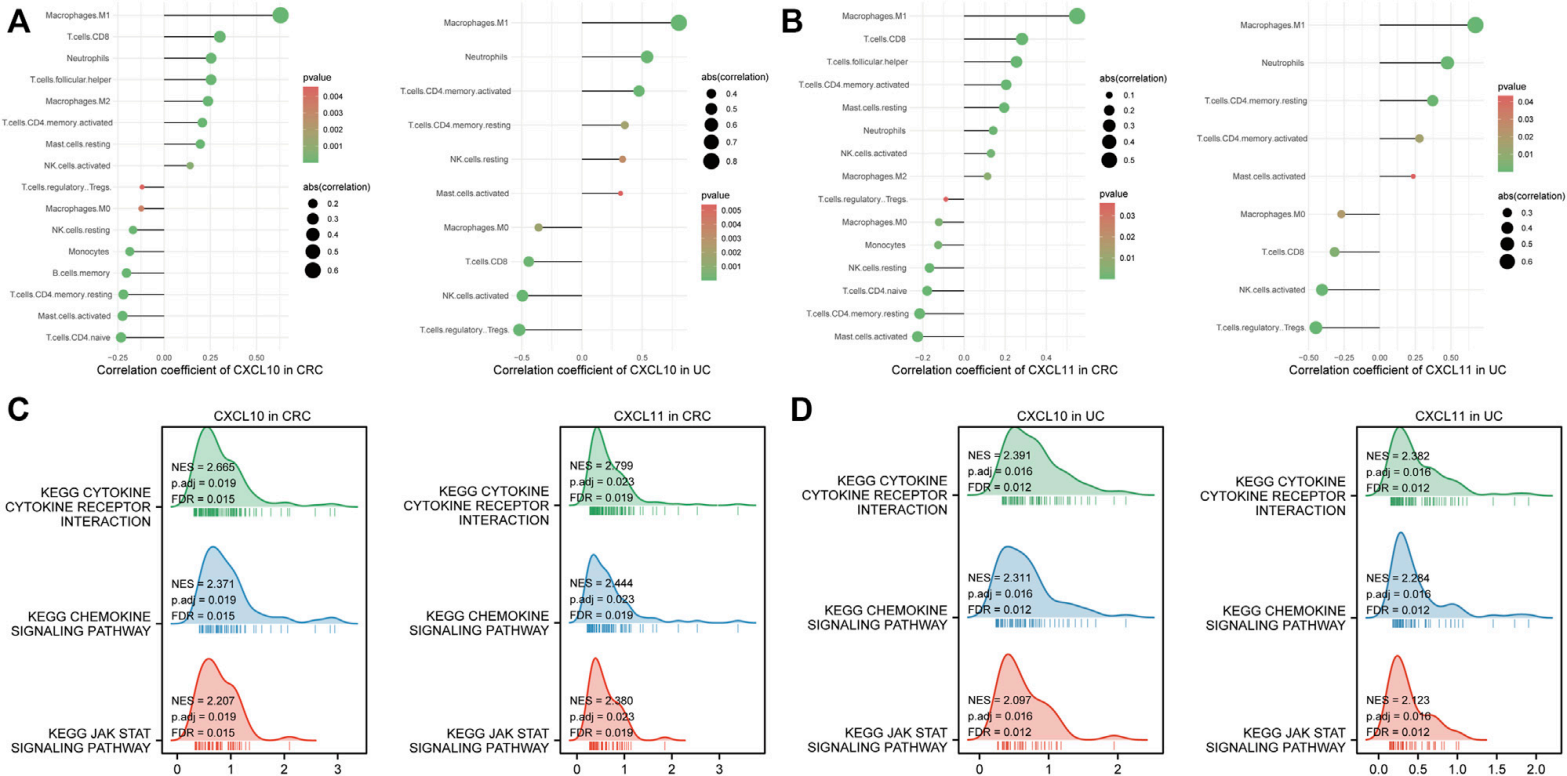
Immune infiltration and gene set enrichment analysis (GSEA) of survival-hub genes. **(A)** correlation analysis between CXCL10 and immune cell infiltration in the Cancer Genome Atlas Program (TCGA) and GSE75214 datasets, respectively. **(B)** correlation analysis between CXCL11 and immune cell infiltration in the TCGA and GSE75214 datasets, respectively. **(C)** GSEA of CXCL10 and CXCL11 in TCGA cohorts. **(D)** GSEA of CXCL10 and CXCL11 in the GSE75214 dataset. Abbreviation: CRC, colorectal cancer; UC, ulcerative colitis; KEGG, Kyoto Encyclopedia of Genes and Genomes; NK, natural killer.

The CIBERSORT algorithm was used to estimate the proportion of 22 immune cells in CRC and UC patients. To explore the correlation between CXCL10/11 and immune infiltration, we further calculated the correlation coefficient of each gene with immune cells. *p*-value < 0.05 was applied to filter significantly correlated immune cells. Figures 7A,B illustrate that CXCL10 and CXCL11 significantly correlated with the infiltration of macrophage M1, neutrophils, CD4^+^ activated memory T cell, and macrophage M0 in CRC and UC cohorts. Moreover, CXCL11 was negatively associated with Tregs infiltration. Moreover, we performed a KEGG pathway analysis of GSEA on CXCL10 and CXCL11 to elucidate the molecular mechanism underlying these two genes. Figures 7C,D consistently show that CXCL10 and CXCL11 might promote the tumorigenesis of CAC through three possible pathways, namely, cytokine–cytokine receptor interaction, chemokine signaling pathway, and JAK–STAT signaling pathway.

## Discussion

Despite advances in therapeutic drugs and cancer screening, UC patients still have a 2.4-fold higher risk of CRC compared with the general population (Jess et al., 2012a). To reduce the incidence of CAC, the shared molecular mechanism between UC and CRC may provide novel insight and targeted molecules to hinder the dysplasia–carcinoma progression for patients with UC. Our results indicated that CXCL10 and CXCL11 might contribute to the tumorigenesis of CAC.

Through bioinformatics analysis and *in vivo* experimentation, we confirmed that CXCL10 and CXCL11 were consistently upregulated in both UC and CRC. Moreover, high expression levels of CXCL10 and CXCL11 were associated with better PFI and early tumor stage in patients with CRC. CXCL10 and CXCL11 were predominantly synthesized and produced by monocytes, endothelial cells, fibroblasts, and cancer cells under the induction of IFN-γ and TNFα (Ohmori et al., 1993; Ohmori et al., 1997). These two cytokines belong to the CXC (C-X-C motif) chemokine family, a group of small secreted proteins attracting and activating immune and nonimmune cells (Réaux-Le Goazigo et al., 2013). CXCR3 is the commonly shared receptor for the activity of CXCL10 and CXCL11. The chronic inflammation underlying UC contributes to the accumulation of inflammatory mediators and immune cells in the intestine, which leads to the increased turnover of epithelial cells, inducing the formation of dysplasia (Rogler, 2014). CXCL10, also known as the interferon *γ*-induced protein-10 (IP-10), has a decisive role in the integrin activation and migration of immune cells (Kuhne et al., 2007). Uguccioni et al. proved that UC patients have significantly higher expression of CXCL10 compared with healthy control in colonic tissues (Uguccioni et al., 1999). Furthermore, the recruitment of the proinflammatory cells mediated by CXCL10 stimulation is responsible for inflammation and tissue damage (Kabashima et al., 2002). Several *in vivo* studies indicated that anti-CXCL10 antibodies could inhibit epithelial ulceration in a UC murine model (Sasaki et al., 2002), attenuate inflammation in IL10^−/−^ mice (Singh et al., 2003), and reduce colitis by compromising T helper type 1 (Th1) induction and recruitment (Hyun et al., 2005). A phase II, randomized, multicenter clinical study has demonstrated the efficacy of monoclonal anti-CXCL10 antibody in moderate-to-severe UC patients who achieved high serum concentrations (Mayer et al., 2014). CXCL11, referred to as interferon-inducible T-cell alpha chemoattractant (I-TAC), could drive Th1 cells to secrete proinflammatory cytokine IL-6 in the inflammatory bowel disease (Liu et al., 2011). One recent study reported that UC patients have significantly higher serum levels of CXCL11 than healthy subjects (Singh et al., 2016). However, there are no reports on the expression levels of CXCL11 in colorectal tissues. Jennifer et al. reported that CXCL11 could also promote tumor progression by activating CXCR7 of tumor cells (Burns et al., 2006). In addition, accumulating evidence has suggested that the CXCL10 and CXCL11/CXCR3 axis could impose antitumor effects by recruiting Th1 cells, cytotoxic T cells, natural killer cells, and natural killer T cells to tumor sites (Hensbergen et al., 2005) and it has protumor effects on cancer cells expressing CXCR3 (Cambien et al., 2009). Taken together, we believe that CXCL10 and CXCL11 may probably involve the malignant transformation of the intestine in patients with UC. Until now, there have been no reported studies on these two genes in the carcinoma pathogenesis of CAC.

MicroRNAs are endogenous noncoding RNAs that could inhibit the expression of genes by specifically binding to the complementary sequences in the 3′-UTR segments of the target mRNAs. After constructing a miRNA–gene interaction network using an online database, we conducted an *in vivo* test to validate the expression difference of three miRNAs in two mouse models. Our results showed that miR-34a-5p and miR-203a-5p were significantly downregulated in both UC and CRC, negatively correlated with CXCL10 and CXCL11. Moreover, Hart et al. discovered that miR-34a-5p could directly inhibit the expression of CXCL10 and CXCL11 by binding to their 3′-UTRs in M1 macrophages (Hart et al., 2020). Thus, low expression of miR-34a-5p may exacerbate the intestine inflammation influenced by UC. Meanwhile, a previous study has suggested that miR-203a-5p acted as a tumor suppressor in CRC (Qian et al., 2019). As a whole, miR-34a-5p and miR-203a-5p have great potential to involve the regulation of CXCL10 and CXCL11 in the tumorigenesis of CAC.

To explore the downstream molecular mechanism mediated by CXCL10 and CXCL11, we used the GSEA method to predict the significantly enriched pathways in the GSE75214 and TCGA datasets, respectively. Our results suggested that CXCL10 and CXCL11 might activate the JAK–STAT signaling pathway via the interaction with cytokine receptors in UC. Most immune regulatory processes are mediated by JAK–STAT signaling, including tumor cell recognition and tumor immune evasion (Owen et al., 2019). Accumulating studies also suggested that the JAK–STAT signaling pathway is critical in promoting chronic inflammation in inflammatory bowel diseases (Egwuagu, 2009). Therefore, CXCL10 and CXCL11 may mediate the tumorigenesis of CAC by activating the JAK–STAT signaling pathway of stromal cells and epithelial cells in the colorectum mucosa.

Immune cells are indispensable components of inflammation in UC and CRC. To elucidate the relationship between CXCL10/11 and immune infiltration, we applied the CIBERSORT algorithm to estimate the proportions of 22 immune cells in the GSE75214 and TCGA datasets. Our results showed that CXCL10 and CXCL11 were positively associated with the infiltration of M1 macrophages in both UC and CRC. Although the M1 macrophage has an antitumorigenic function, in chronic inflammation, it could induce more severe inflammation by secreting proinflammatory cytokines and reactive oxygen species (Chen et al., 2021).

The primary limitation of this study is the lack of available datasets consisting of matched CAC, UC, and corresponding normal groups, which may comprise the validity of our predicted genes. However, Zhao et al. reported that CAC and non-UC-associated CRC patients have a high degree of similarity in gene expression (Zhao et al., 2013). On the other hand, our study conducted comprehensive bioinformatics analysis and *in vivo* experiments to predict and validate potential genes involved in the tumorigenesis of CAC.

## Conclusion

Our study demonstrated that CXCL10 and CXCL11 might participate in the tumorigenesis of CAC by mediating the chronic inflammation in UC. Targeting CXCL10 and CXCL11 could be a promising therapeutic strategy to prevent CAC development in patients with UC.

## Data Availability

The original contributions presented in the study are included in the article/Supplementary Material. Further inquiries can be directed to the corresponding author.

## Supplementary material

Supplementary Table S1 Sequences of primers used in the experiment.

Supplementary Table S2 Gene ontology and Kyoto Encyclopedia of Genes and Genomes enrichment analyses of common DEGs.

Supplementary Table S3 Characteristics of patients with colorectal cancer in the Cancer Genome Atlas Program cohorts.
