# Supplementary material for: Identification of CXCL10 and CXCL11 as the candidate genes involving the development of colitis-associated colorectal cancer
Source: Front Genet. 2022 Aug 8;13:945414. doi: 10.3389/fgene.2022.945414 (PMC9393335; doi:10.3389/fgene.2022.945414)
Supplement: Supplementary file 1 [file Table1.DOCX]

<https://www.jianguoyun.com/p/DS5n-r0QvJDOChjmq8IEIAA>
