# Supplementary figures and images for: Identification of CXCL10 and CXCL11 as the candidate genes involving the development of colitis-associated colorectal cancer
Source: Front Genet. 2022 Aug 8;13:945414. doi: 10.3389/fgene.2022.945414 (PMC9393335; doi:10.3389/fgene.2022.945414)

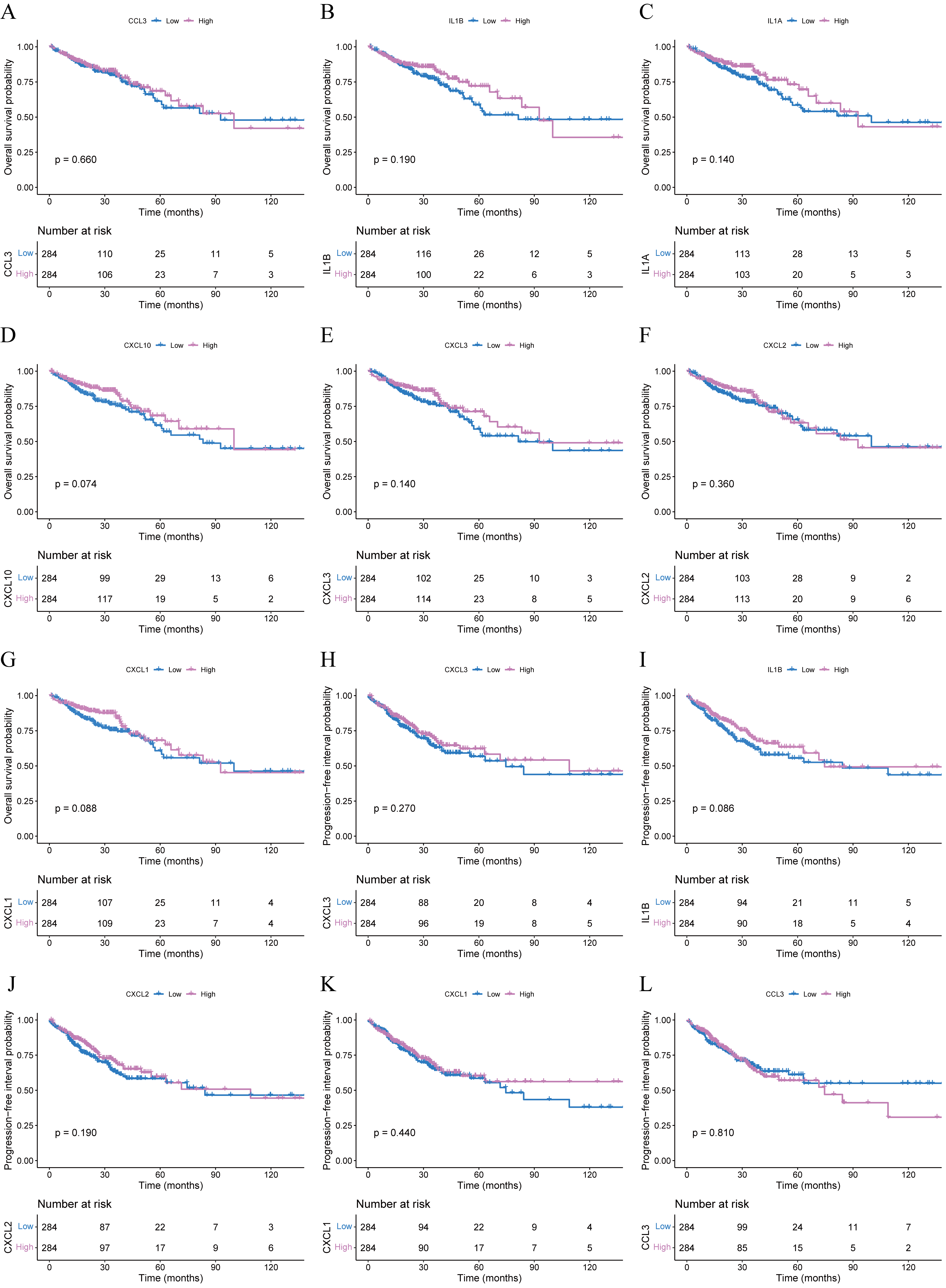

Supplement: Supplementary file 2 [file image1.tif]
